# Constraints and Adaptation of Closed-Loop Neuroprosthetics for Functional Restoration

**DOI:** 10.3389/fnins.2017.00111

**Published:** 2017-03-13

**Authors:** Robert Bauer, Alireza Gharabaghi

**Affiliations:** Division of Functional and Restorative Neurosurgery, Centre for Integrative Neuroscience, Eberhard Karls University TuebingenTuebingen, Germany

**Keywords:** assistive technology, neurorehabilitation, stroke, rehabilitation robotics, brain-computer interface, brain-machine interface, brain-robot interface

## Abstract

Closed-loop neuroprosthetics aim to *compensate* for lost function, e.g., by controlling external devices such as prostheses or wheelchairs. Such assistive approaches seek to maximize speed and classification accuracy for high-dimensional control. More recent approaches use similar technology, but aim to restore lost motor function in the long term. To achieve this goal, *restorative* neuroprosthetics attempt to facilitate motor re-learning and to strengthen damaged and/or alternative neural connections on the basis of neurofeedback training within rehabilitative environments. Such a restorative approach requires reinforcement learning of self-modulated brain activity which is considered to be beneficial for functional rehabilitation, e.g., improvement of β-power modulation over sensorimotor areas for post-stroke movement restoration. Patients with motor impairments, however, may also have a compromised ability for motor task-related regulation of the targeted brain activity. This would affect the estimation of feature weights and hence the classification accuracy of the feedback device. This, in turn, can frustrate the patients and compromise their motor learning. Furthermore, the feedback training may even become erroneous when unconstrained classifier adaptation—which is often used in assistive approaches—is also applied in this rehabilitation context. In conclusion, the conceptual switch from assistance toward restoration necessitates a methodological paradigm shift from classification accuracy toward instructional efficiency. Furthermore, a constrained feature space, a priori regularized feature weights, and difficulty adaptation present key elements of restorative brain interfaces. These factors need, therefore, to be addressed within a therapeutic framework to facilitate reinforcement learning of brain self-regulation for restorative purposes.

## Restoration instead of assistance

Brain self-regulation has recently been applied in the context of motor rehabilitation after stroke by providing contingent feedback of motor imagery (Buch et al., 2012; Ang et al., 2014; Morone et al., 2015; Pichiorri et al., 2015). In these approaches, specific brain states (i.e., rest vs. motor imagery) are often separated using an online analysis of sensorimotor power in a cue-paced trial-structure. When used in conjunction with robotic rehabilitation technology, these devices are also referred to as brain-robot interfaces (BRI; Bauer et al., 2015; Naros and Gharabaghi, 2015; Kraus et al., 2016).

While *assistive* BRIs aim to replace lost function by controlling external devices (Hochberg et al., 2012; Collinger et al., 2013), *restorative* BRIs aim to rehabilitate an impaired function (Gharabaghi, 2016; Krucoff et al., 2016). In such a restorative framework, BRIs adhere to an operant conditioning rationale (Sherlin et al., 2011; Bauer and Gharabaghi, 2015b). They provide contingent feedback to facilitate the self-regulation of specific brain activity. This reinforcement learning-based approach is considered to be beneficial for recovery and might ultimately lead to functional gains on the basis of motor re-learning and strengthening of damaged and/or alternative neural connections (Daly and Wolpaw, 2008). Restorative BRIs might be additionally supported by brain state dependent stimulation to strengthen cortico-spinal connectivity (Gharabaghi et al., 2014a; Royter and Gharabaghi, 2016; Kraus et al., 2016).

## Methodological adjustments

We propose that, on account of their different goals, these restorative techniques require a different methodological approach than assistive BRIs, i.e., modifying brain physiology vs. controlling extremal devices. We acknowledge that different strategies may be adopted to achieve modified neurophysiology and, ultimately, behavioral gains. However, on the basis of empirical evidence acquired in our lab, we propose the following adjustments: constrained feature space, regularized feature weights, and difficulty adaptation.

Instead of analyzing all acquired signals for optimal classification, we propose that the feature space be intentionally constrained to reinforce a specific oscillatory pattern in accordance with the respective treatment rationale (*constrained feature space*). In a next step, to differentiate between the classes, assistive BRIs use classifier calibration to weight features according to their relevance. However, learning brain self-regulation may lead to non-stationarity of these classes in the course of the training (Vidaurre et al., 2011a; Sugiyama et al., 2013; Naros and Gharabaghi, 2015). Unsupervised adaptation of the feature weights may therefore lead to a switch in the mental strategy (Vidaurre et al., 2011b; Bryan et al., 2013). This approach may even result in artefactual control (Gharabaghi et al., 2014b). We, therefore, propose that *feature weight regularization* be applied to address this issue. Furthermore, cognitive, sensory, and motor impairments may limit the ability to modulate brain activity, perceive, and/or process feedback. This may cause frustration, which, in turn, may be exacerbated due to the low classification accuracy caused by the constrained and regularized feature space (Nijboer et al., 2008; Fels et al., 2015). In this context, we propose that *difficulty adaptation* be applied to overcome cognitive load issues (Bauer and Gharabaghi, 2015a; Bauer et al., 2016a,b). Such an approach may also improve the instructional efficiency of feedback (Bauer and Gharabaghi, 2015b) and maintain motivation (Bauer et al., 2016a,b).

In the following paragraphs, we discuss these methodological adjustments in greater detail.

## Constrained feature space

In high-dimensional feature spaces, some regions may be sparsely populated with data, thereby, impairing the classifier setup (Theodoridis and Koutroumbas, 2009). Under these circumstances, constraining the feature space provides a way of dealing with this *curse of dimensionality*. However, if the feature space is constrained a priori, some useful features for classification may also be discarded. A classifier based on a constrained feature set therefore usually performs less well than a classifier based on a full feature set.

Restorative BRIs, which apply this approach, therefore appear inferior in comparison to their assistive counterparts. The latter use more flexible algorithms to select and weight all available features and to maximize classification accuracy (Ang et al., 2009; Theodoridis and Koutroumbas, 2009). An a priori constraint should therefore be well considered. It is tempting to assume that the brain will find the best combination of features by itself. Such an approach is therefore implicitly followed during standard or robotic neurorehabilitation, when the feedback that is provided by the therapist or the training device is independent of specific brain features. This strategy, however, has not been successful until now, at least when considering severely motor-impaired stroke patients with persistent deficits. Moreover, the features (α-desynchronization) identified as most useful for classification between different states in the post-stroke brain, e.g., rest vs. motor imagery, are not necessarily those that are most therapeutically relevant (β-desynchronization): Synchronization/Desynchronization describe the (often task-induced) increase/reduction in power in specific frequency bands. The α-band usually ranges from 8–14 Hz, while the β-band ranges from 15–30 Hz. Specifically, movement-related β-desynchronization (β-ERD) is compromised in the contralateral primary cortex in comparison to healthy controls; the more severe the patient's motor impairment, the less β-ERD (Rossiter et al., 2014). And so β-ERD remains inferior to other features for classification purposes in stroke patients, e.g., in differentiating movement-related brain states for the control of external devices (Gomez-Rodriguez et al., 2011). In this context, we argue that the fact that β-oscillations are less optimal for classification purposes does not compromise—but rather qualifies—this physiological marker as a therapeutic target (Naros and Gharabaghi, 2015). Here, we see an analogy to the concept of constraint-induced movement therapy in stroke patients, where the affected rather than the healthy side of the body is trained to facilitate restoration instead of compensation of motor function. Notably, such an approach does not exclude the possibility that alternative cortico-spinal pathways which do not originate from the contralateral primary motor cortex take over lost function. These pathways would be facilitated on the basis of cortical disinhibition and coherent interaction with the muscles in the β-band as well (Mima et al., 2001; Kilavik et al., 2012; Aumann and Prut, 2015; Brittain et al., 2014; Rossiter et al., 2014; Kraus et al., 2016).

Furthermore, an approach based on a constrained feature space allows making a direct, hypothesis-driven comparison of different interventions based on specific oscillatory patterns. By way of example, an increase in the β-modulation range will improve cortico-spinal connectivity (Kraus et al., 2015, 2016) and motor function (Naros et al., 2016). This will enable us to empirically detect functionally relevant markers and mechanisms of restoration and to determine physiology-based strategies for further improvement. Such knowledge will also enable us to develop approaches for treatment matching, e.g., defining feature sets on the basis of specific functional impairments and/or lesion locations (Shelton and Reding, 2001; Stinear et al., 2012). By contrast, an approach based on an unconstrained feature space would be based on the assumption that the most accurate detection of motor intention/imagery and provision of feedback is in itself sufficient to restore function.

## Regularized feature weights

Regularization can be considered a penalty term to prevent feature weights from reaching implausibly high values (Theodoridis and Koutroumbas, 2009; Bishop, 2013) caused by the empirical estimation of class parameters (e.g., mean and covariance). Such estimates can be biased, especially when the sample size is low. Due to the large variety of classification approaches (Theodoridis and Koutroumbas, 2009), several regularization approaches have been suggested, e.g., pooled covariance estimation (Friedman, 1989), rejection of eigenvectors (Blankertz et al., 2008), shrinkage estimators (Beltrachini et al., 2010), or feature subset selection (Friedman, 1989).

Even when recognizing that a constrained feature space can already be considered a form of regularization, the empirical determination of feature weights during the calibration period may pose a particular challenge for restorative brain-interface approaches, e.g., when estimating mean and covariance of two classes (rest vs. motor imagery). When patients are able to desynchronize sensorimotor oscillations (Pfurtscheller et al., 2005; Neuper et al., 2006; Kaiser et al., 2011), the estimation of feature weights is usually straightforward. In such a case, several approaches for regularization have been discussed (Yuan and Bentler, 1998; Beltrachini et al., 2010). If, however, the volitional modulation of sensorimotor oscillations has not been learned (Brauchle et al., 2015; Bauer and Gharabaghi, 2015b; Naros and Gharabaghi, 2015), or when it is impaired due to the underlying pathology (Buch et al., 2012; Bundy et al., 2012; Rossiter et al., 2014), the estimation might become noisy or even false. More formally, if one class (i.e. motor imagery) is not sufficiently expressed, its parameters (e.g., mean and covariance) cannot be measured. If, however, the mean during motor imagery is not sufficiently different from the mean during rest, a noisy estimate can result in the classifier being calibrated toward the wrong direction of modulation. Subsequently, the patient might receive feedback for synchronizing instead of desynchronizing.

Novelty detection has been suggested as a solution, if no information about a second class is available (Pimentel et al., 2014). Such a one-class approach might base mean and covariance estimation on the rest class only. However, without a priori information about the targeted direction of modulation, data-driven regularization approaches cannot be sufficient.

Furthermore, when a patient alters the mental strategy in the course of the intervention, a classifier trained on the initial strategy can become misaligned. In classical brain-interface approaches, the adaptation of feature weights has been proposed for such cases (Vidaurre et al., 2011b; Bryan et al., 2013; Sugiyama et al., 2013). But such data-driven approaches can be problematic for restorative approaches; classifier adaptation might condition the patients to explore alternative, i.e., therapeutically non-desired strategies (Bauer and Gharabaghi, 2015a,b). When the patient becomes frustrated with motor imagery, he/she may use artifacts for control, e.g., muscle contractions (Gharabaghi et al., 2014b).

Bearing these points in mind, we suggest employing informed regularization determined by a priori selected feature weights, thereby, ensuring the targeted direction of modulation. In our lab, we currently employ a variant of novelty classification by using a linear discriminant analysis with a fixed direction. In that regard, we base the mean and covariance estimation on the rest class only, with the parameter estimation pooled across several electrodes. Thereby, we provide feedback for the reduction of the mean, i.e., desynchronization, only.

## Difficulty of adaptation

Lotte and colleagues have pointed out that most neurofeedback protocols are limited with regard to their instructional design. They suggested adaptive training approaches, i.e., the use of difficulty levels which are challenging, but still achievable (Lotte et al., 2013). A similar idea was postulated by the cognitive load theory (Schnotz and Kürschner, 2007). On the basis of these concepts, both under- and over-challenge must be avoided to facilitate learning (Schnotz and Kürschner, 2007; Bauer and Gharabaghi, 2015a). In classifiers, which are constrained, regularized and linear, item response theory enables us to directly relate the threshold used for classification to the difficulty level (Bauer and Gharabaghi, 2015a). By using a linear discriminant analysis with a fixed direction, thresholding allows us to provide reward for desynchronization only when it is sufficiently strong. Within this framework, the shape of classification accuracy CA across different threshold can be interpreted as the zone of proximal development (ZPD). This argument, with detailed examples, has been clarified elsewhere (Bauer and Gharabaghi, 2015a). The ZPD is an indirect measure of a subject's cognitive resources (Schnotz and Kürschner, 2007). It also constitutes the range of threshold, where learning may occur because subjects are able to compensate for the extraneous load caused by the mismatch of ability and difficulty (Bauer and Gharabaghi, 2015a). Along these lines, two recent studies with healthy subjects provided empirical evidence that dynamic threshold adaptation is instrumental in facilitating learning (Bauer et al., 2016b; Naros et al., 2016).

Unconstrained and unregularized classifiers do not offer an accessible, one-dimensional parameter to fine-tune the difficulty of the task. It might therefore be problematic to adapt the difficulty within these approaches. In particular, a multi-dimensional or even non-linear theory of difficulty adaptation appears to be challenging. We instead explored the difficulty threshold of a linear, a priori constrained and regularized classifier and found evidence of a direct correlation between the subjects' perceived mental effort and the task difficulty (Bauer et al., 2016b). Further empirical evidence suggests that there is a link between classification accuracy and cognitive load; classification performance has been linked to mood and mastery confidence (Nijboer et al., 2008), as well as to the degree of concentration on the task and the ability to ignore distracting stimuli (Hammer et al., 2012). The sensation of challenge might therefore be linked to the ratio of true to false positives returned by the classifier. This hypothesis is supported by a Bayesian simulation study of reinforcement learning under adaptive changes of true and false positive rates (Bauer and Gharabaghi, 2015b). A generalized concept of difficulty adaptation might, therefore, be based on controlling the relationship between true and false positive rates by asking the patients to self-rate the perceived effort and/or applying non-cued training.

Nonetheless, further factors may affect the difficulty of the training: the challenge of achieving a sense of cognitive and internal control (Burde and Blankertz, 2006; Wood et al., 2014), the appropriate processing of cues to reduce impairments in mental chronometry (Liepert et al., 2012), and to increase the quality of motor imagery (Heremans et al., 2009, 2012), the specific sensory impairments of patients and their interaction with the feedback modality (e.g., visual, haptic, auditory) (Nijboer et al., 2008; Gomez-Rodriguez et al., 2011; Parker et al., 2011; Sollfrank et al., 2015), or the repetitive and fatiguing nature of training (Lee et al., 1991; Page et al., 2011). Dealing with these aspects by proper instructional design is more important for restorative than for assistive approaches (Lotte et al., 2013).

## Conclusion

We propose that restorative approaches should apply prior information about beneficial features (e.g., β-power desynchronization over sensorimotor areas) to constrain the feature space and regularize their direction. Such an approach may reduce the classification accuracy in comparison to unconstrained or unregularized approaches, particularly in patients who are only partially able to self-regulate the targeted brain state. At the same time, this method would increase the likelihood that feedback is provided for the therapeutically targeted modulation of brain activity only. The threshold selection in restorative approaches should therefore not be misled by the goal of maximum classification accuracy. Instead, it should follow instructional demands to maximize learning.

Accordingly, several methods have been proposed for locating the threshold for maximum learning (Ivanova et al., 2005; Cegarra and Chevalier, 2008; Naros et al., 2016; Bauer et al., 2016a,b). Moreover, physiological parameters, e.g., distributed cortical patterns in the α-range (Vukelić et al., 2014; Vukelić and Gharabaghi, 2015a,b) and the θ-range (Fels et al., 2015) which were linked to β-band self-regulation may also be used in the long term for this purpose.

The conceptual switch from assistive to restorative neuroprosthetics necessitates methodological adjustments (constrained feature space, a priori regularized feature weights, difficulty adaptation) which ultimately represent a paradigm switch from classification accuracy toward instructional efficiency to facilitate reinforcement learning of brain self-regulation.

## Author contributions

All authors listed, have made substantial, direct and intellectual contribution to the work, and approved it for publication.

### Conflict of interest statement

The authors declare that the research was conducted in the absence of any commercial or financial relationships that could be construed as a potential conflict of interest.
